# Attitudes of Pediatricians Regarding Prevention and Treatment of Vitamin D Deficiency

**DOI:** 10.4274/jcrpe.2978

**Published:** 2016-09-01

**Authors:** Gülcan Seymen Karabulut, Şükrü Hatun, Aysun Bideci, Enver Hasanoğlu

**Affiliations:** 1 Kocaeli University Faculty of Medicine, Department of Pediatric Endocrinology, Kocaeli, Turkey; 2 Gazi University Faculty of Medicine, Department of Pediatric Endocrinology, Ankara, Turkey; 3 National Pediatrics Association, Ankara, Turkey

**Keywords:** Vitamin D deficiency, prevention, pediatrician attitudes

## Abstract

To determine the adherence of pediatricians to the nationwide ‘Vitamin D Prophylaxis Program’ and to evaluate their attitudes about vitamin D intake. The study was conducted using the Turkish National Pediatrics Association network. The pediatricians were asked to respond to an online questionnaire that included five questions on ‘What dose of vitamin D they recommend for supplementation?’, ‘At what age they start vitamin D supplementation?’, ‘Supplementation method’, ‘Clichés and truths about vitamin D’, and ‘High-dose vitamin D therapy indications’. Responses of 167 pediatricians were evaluated in this study. 75.5% of pediatricians indicated that they recommended vitamin D supplementation in a daily dose of 400 IU. 47.1% started vitamin D supplementation by the end of the 2^nd^ week. 7.83% of pediatricians suggested doubling the daily dose of vitamin D supplementation in infants with delayed tooth eruption, 19.9% suggested immediate cessation of vitamin D supplementation in infants with small anterior fontanels. This study showed that the majority of the pediatricians still prescribe vitamin D prophylaxis late, recommend high doses of vitamin D in cases of delayed tooth eruption, and think that low serum 25-hydroxy vitamin D level regardless of alkaline or phosphatase parathyroid hormone measurement is an indication for high-dose vitamin D (stoss) therapy. These results suggest a need for new training programs focusing on vitamin D supplementation.

## INTRODUCTION

While there is an increased awareness on the critical role of vitamin D on health, vitamin D deficiency is still an important health problem due to the influence of social, cultural, and geographic factors (1). Symptomatic vitamin D deficiency leads to morbidities including congenital and infantile rickets, hypocalcemic convulsions, dilated cardiomyopathy, skeletal myopathy, and osteomalacia from fetal life to adulthood. It may even lead to death. All these conditions are reversible and also preventable. Therefore, it is essential to clarify the responsibilities of families, physicians, and healthcare system in preventing vitamin D deficiency and to establish vitamin D supplementation as a public health priority similar to vaccination (2).

A nationwide vitamin D prophylaxis program that included free distribution of vitamin D drops to all newborns and infants (0-12 months) attending primary health centers throughout the country was initiated in 2005 in Turkey (3). However, it has been reported that physician attitude for vitamin D supplementation is not always in compliance with guidelines and also that some parents are unwilling to keep up with recommendations (4,5,6).

This study aimed to determine the adherence of pediatricians to the guidelines of the program and to evaluate their fundamental attitudes about vitamin D deficiency and vitamin D supplementation.

## METHODS

The study was conducted making use of the Turkish National Pediatrics Association Network. The questionnaire was sent to 1800 pediatricians. The pediatricians filled out an online questionnaire regarding their policy on vitamin D supplementation.

The questionnaire consisted of 5 questions. The first part three questions were on vitamin D supplementation practices:

What daily dose of vitamin D supplementation do you recommend? 1200 IU, 400 IU, 600 IU, 800 IU.

At what age do you recommend to start vitamin supplementation? Soon after birth, at the end of the 1st week, at the end of the 2nd week, at the end of the 3rd week, after age one month.

Which method do you prefer for vitamin D supplementation? Three drops orally once daily (400 IU/day), eight drops orally once daily (1000 IU/day), one vial (300.000 IU) orally once a month, one vial (300.000 IU) orally every two months, one vial (300.000 IU) intramuscularly every two months.

We also asked whether their practices were influenced by some common non-scientific beliefs.

Clichés and truths about vitamin D intake (giving double dose of daily vitamin D prophylaxis in infants with delayed tooth eruption, abstaining from vitamin D supplementation in infants with small anterior fontanelles, giving extra doses of vitamin D to infants with delayed walking and/or leg bowing).

The last question queried whether they followed the under-mentioned indications for high-dose vitamin D therapy (stoss therapy) in their clinical practice.

High-dose vitamin D therapy indications for all newborns and infants consist of a 25-hydroxy vitamin D [25(OH)D] level <15 ng/mL, an elevated serum alkaline phosphatase levels in addition to a serum 25(OH)D level of <15 ng/mL, and presence of craniotabes in addition to a serum 25(OH)D level <15 ng/mL.

## RESULTS

A total of 167 pediatricians completed the questionnaire. 75.5% of them indicated that they routinely recommended daily vitamin D prophylaxis in a dose as 400 IU. 10.2% of respondents recommended vitamin D prophylaxis in a daily dose of 800 IU. The remainder of the responders stated they recommended daily doses of 1200 IU and 600 IU. 28.7 % of respondents recommended beginning vitamin D prophylaxis soon after birth and 47.3% recommended beginning by the end of the 2nd week. 86.2% of respondents recommended daily 3 drops for vitamin D prophylaxis, while 11.38 % recommended daily 8 drops and 1.2% administration of oral 300.000 IU every 2 months.

7.8% of respondents suggested doubling the daily dose of vitamin D prophylaxis in infants with delayed tooth eruption, 19.9% suggested immediate cessation of vitamin D in infants with small anterior fontanelle, while 2.4% suggested giving 300.000 IU of vitamin D every two months to infants with delayed walking and 16.9% suggested giving 300.000 IU of vitamin D to infants with leg bowing (Table 1).

## DISCUSSION

The nationwide vitamin D prophylaxis program of the Turkish Ministry of Health successfully reduced the prevalence of rickets in Turkey from 6% in 1998 to 0.1% in 2008 in children under 3 years of age (3). This program is closely controlled by the Turkish Ministry of Health using a ‘performance assessment system’ that monitors primary care doctors’ practices and rewards those who are compliant with the nationwide vitamin D prophylaxis program. In this program, the recommended daily vitamin D dose is 400 IU, which is compatible with the “Institute of Medicine (IOM) Committee’s http://www.nationalacademies.org/hmd/ 2011 Report on Dietary Reference Intakes for Calcium and Vitamin D”. This dose was chosen also because a daily 400 U supplementation of vitamin D was shown to be adequate to provide a serum 25(OH)D level >15 ng/mL in >90% of all infants (7,8). In a large scaled survey study by the Ministry of Health to evaluate the success of the nationwide program, it was shown that of 2504 infants aged between 6 and 17 months, serum 25(OH)D level was >15 ng/mL in 73.6% (9). This is consistent with our survey findings that a similar percentage of pediatricians adhere to recommended vitamin D supplementation of the nationwide program. The survey also showed that a great majority of pediatricians preferred giving vitamin D supplementation daily and orally.

While this was encouraging, more than half of the pediatricians who responded to our questionnaire recommend to start supplementation later than the age recommended by the Ministry of Health. We believe this finding is mostly due to the misconception among pediatricians that the maternal transfer of vitamin D in utero would protect the infants against vitamin D deficiency in the first 3 weeks of life (10). However, because of the high incidence of maternal vitamin D deficiency, the American Academy of Pediatrics determined the beginning time of vitamin D supplementation as the first few days of life (6,11,12). We believe that a new information campaign is needed to change the physicians’ attitudes for the starting age of vitamin D prophylaxis.

The results of our study reveal that non-scientific beliefs may influence clinical practice. 20% of the physicians thought that vitamin D supplementation should be stopped in babies with small anterior fontanels. We suppose that this belief is because of a misconception that if vitamin D deficiency can delay anterior fontanel closure, an early closure of fontanel should indicate vitamin D excess. Another stereotyped approach is erroneously considering bowing in infants automatically as a sign of vitamin D deficiency and prescribing high doses of vitamin D without additional findings though it may be due to other causes (physiological, skeletal dysplasia, or hypophosphatemic rickets). Similarly, 10% of pediatricians have stated that high-dose vitamin D treatment will accelerate tooth eruption and walking. This is consistent with a common belief among the parents.

Another interesting finding is that most pediatricians routinely analyze serum 25(OH)D and prescribe high doses of vitamin D (stoss therapy) without assessing the clinical symptoms and/or other biochemical and radiological parameters. We believe this attitude is due to the widely publicized extraskeletal effects of vitamin D (13). However, recently, treatment of vitamin D deficiency rickets has been revised and “stoss” or “single-dose” therapy dosage reduced to 50 000 units for the first year of life (14). It is also important to note that in Turkey, 300.000 IU vitamin D vials are available without prescription. The pediatricians’ attitude as well as the common belief among the parents that high-dose vitamin D should be given to those children with delayed teething and/or walking, have led to a possible increase in Turkey of cases of vitamin D intoxication (15,16). In order to prevent vitamin D intoxication, the pediatricians need to be educated on this matter and over-the-counter sale of high-dose vitamin D preparations should be forbidden.

In conclusion, despite significant progress in the prevention and treatment of vitamin D deficiency in Turkey, some pediatricians still have incorrect attitudes such as starting vitamin D supplementation late, using high doses of vitamin D without a real indication, and accepting low serum 25(OH)D levels sufficient to begin high-dose vitamin D therapy. We believe there is a need for a new reinforcing education program among the pediatricians.

## Ethics

Peer-review: Externally peer-reviewed.

## Figures and Tables

**Table 1:**
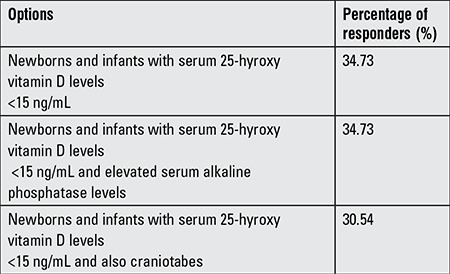
For which of the following groups of infants is high-dose vitamin D therapy most appropriate?
